# Diagnosis of Esophageal Lesions by Multi-Classification and Segmentation Using an Improved Multi-Task Deep Learning Model

**DOI:** 10.3390/s22041492

**Published:** 2022-02-15

**Authors:** Suigu Tang, Xiaoyuan Yu, Chak-Fong Cheang, Zeming Hu, Tong Fang, I-Cheong Choi, Hon-Ho Yu

**Affiliations:** 1 Faculty of Information Technology, Macau University of Science and Technology, Macau 999078, China; 2009853gii30001@student.must.edu.mo (S.T.); 1709853eii30001@student.must.edu.mo (X.Y.); 1909853gii20004@student.must.edu.mo (Z.H.); 19098533ii20002@student.must.edu.mo (T.F.); 2 Kiang Wu Hospital, Macau 999078, China; ecg@kwh.org.mo (I.-C.C.); yuhonho@kwh.org.mo (H.-H.Y.)

**Keywords:** classification, deep learning, esophageal lesions, gastrointestinal endoscopy, multi-task, segmentation

## Abstract

It is challenging for endoscopists to accurately detect esophageal lesions during gastrointestinal endoscopic screening due to visual similarities among different lesions in terms of shape, size, and texture among patients. Additionally, endoscopists are busy fighting esophageal lesions every day, hence the need to develop a computer-aided diagnostic tool to classify and segment the lesions at endoscopic images to reduce their burden. Therefore, we propose a multi-task classification and segmentation (MTCS) model, including the Esophageal Lesions Classification Network (ELCNet) and Esophageal Lesions Segmentation Network (ELSNet). The ELCNet was used to classify types of esophageal lesions, and the ELSNet was used to identify lesion regions. We created a dataset by collecting 805 esophageal images from 255 patients and 198 images from 64 patients to train and evaluate the MTCS model. Compared with other methods, the proposed not only achieved a high accuracy (93.43%) in classification but achieved a dice similarity coefficient (77.84%) in segmentation. In conclusion, the MTCS model can boost the performance of endoscopists in the detection of esophageal lesions as it can accurately multi-classify and segment the lesions and is a potential assistant for endoscopists to reduce the risk of oversight.

## 1. Introduction

Esophageal diseases are one of the most common diseases in humans, resulting in threatening to human health such as esophageal cancer. In 2020, the number of incidents ranked tenth in the world, with 604,100 new cases, and the number of deaths ranked sixth, with 544,076 deaths [1]. When treating cancer, the 5-year survival rate of early esophageal cancer patients is higher than 90%, and the 5-year survival rate of advanced esophageal cancer patients is lower than 20% [2]. Therefore, it is important to diagnose various esophageal lesions quickly and accurately.

Generally, gastrointestinal endoscopic screening has become the typical diagnostic choice for the evaluation of patients with esophageal diseases. Unfortunately, it is sometimes difficult to accurately distinguish some esophageal lesions, such as normal and esophagitis, or esophagitis and cancer, based on conventional endoscopic white-light imaging (WLI) because of its lower sensitivity and specificity [3]. Narrow-band imaging (NBI) is an advanced imaging system that overcomes the drawbacks of WLI, but it extends examination time and requires experienced endoscopists [4]. Moreover, endoscopists with less experience are unlikely to be able to differentiate similar esophageal lesions, because of their visual similarity in terms of shape, size, and texture. Additionally, endoscopists require to carefully interpret a larger lot of esophageal images one by one to make the correct diagnosis every day. Therefore, developing an effective computer-aided diagnostic tool is of great significance to reduce the burden of endoscopists in analyzing esophageal lesions.

Lesion classification and lesion segmentation are the two basic tasks of computer-aided diagnostic tools to help endoscopists to formulate reliable diagnosis schemes based on analyses of classification and segmentation for esophageal lesions. Lesion classification can help endoscopists quickly distinguish different types of lesions from a large number of endoscopic images, saving a lot of time. Lesion segmentation can further annotate the shape, size, and texture of lesions, which is very important for the clinic. Previous methods of medical lesion classification and segmentation often depended on support vector machine [5], template matching techniques [6], active contours [7], edge detection [8], and so on. However, these approaches depend on the utilization of hand-crafted features. It is difficult to design representative features for different applications, as the features designed for one type of medical image can only be used for this specific type, but often fail in other types. Therefore, a general approach to extracting the features is lacking.

With the rapid development of convolutional neural networks in image processing, an increasing number of deep learning-based methods have been proposed and have achieved obvious success in different medical image analyses such as diabetes prediction [9,10], cervical cancer detection [11], and skin disease classification [12]. For esophageal lesions analyses, a large number of studies have been conducted on the diagnosis of esophageal diseases by endoscopy based on the deep learning model, which has shown better performance than traditional methods [13,14,15]. For example, Liu et al. [16] used a convolutional neural network consisting of O-stream and P-stream to classify esophageal cancer and premalignant lesions with 85.83% accuracy and 94.23% sensitivity. Du et al. [17] proposed an efficient channel attention deep dense convolutional neural network to divide esophageal lesions into four categories and achieved an accuracy of 90.63%. Wang et al. [18] put forward a multi-scale context-guided deep network to segment esophageal lesions with high mean intersection over union. More research about deep learning in esophageal lesion analysis is summarized in Table 1.

Although these deep learnings have achieved obvious success in the classification or segmentation of esophageal lesions, they have a common problem: they are only used for a single task. The classification and segmentation of esophageal lesions provide comprehensive information for endoscopists to fully understand the status of esophageal lesions and are indispensable for computer-aided diagnostic tools. Hence, we propose a multi-task classification and segmentation (MTCS) model, incorporating an Esophageal Lesions Classification Network (ELCNet) and an Esophageal Lesions Segmentation Network (ELSNet), to realize the classification and segmentation of esophageal lesions using esophageal endoscopic images. The ELCNet was used to classify esophageal lesions into three categories: cancer, esophagitis, and normal, and when the image is predicted as esophageal cancer, the ELSNet can locate the lesion area. More importantly, in order to achieve an accurate diagnosis, especially when there are differences in the size and shape of the same type of lesions, it is essential to allow the deep neural network to learn the best representative features of lesions. However, these professional skills are usually acquired only by experienced endoscopists who are capable of a detailed examination of the core features of subtle differences among lesions. Therefore, inspired by [27], we used dilated convolution to improve the proposed deep learning model by enlarging the receptive field in convolutional neural networks, thereby allowing the model to extract the most useful features of the esophageal images when training. The proposed model can assist endoscopists to diagnose esophageal lesions in an efficient strategy while reducing labor and misdiagnosis as much as possible.

The rest of this paper is organized as follows. In Section 2, we introduce materials and methods in detail. Section 3 shows experiments and results. Discussion is presented in Section 4. Finally, we get the conclusions in Section 5.

## 2. Materials and Methods

### 2.1. Training and Validation Sets

**Figure 1:**
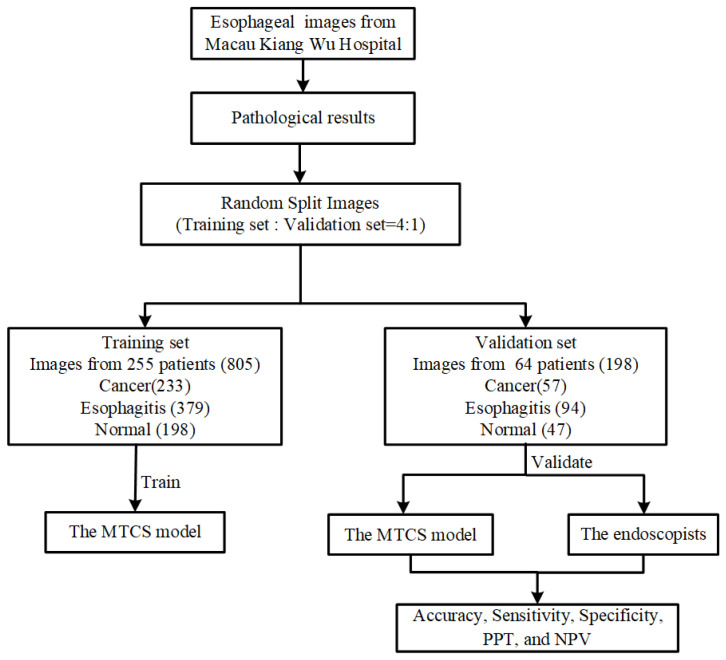
Workflow diagram for the training set and validation set of the MTCS model.

This study was conducted at the most representative hospital in Macau, Kiang Wu Hospital, which is the largest private hospital and has a maximum quantity of cases and patients of esophageal diseases in Macau. We retrospectively collected 1003 esophageal images from 319 patients, captured during screening or preoperative examinations in daily clinical practice between 2016 and 2019. The abnormal esophageal images included lesions of cancer and esophagitis. All these images were randomly divided into a training set and a validation set according to a 4:1 ratio. The training set was used to train the MTCS model and included a total of 805 images from 255 patients, consisting of 233 cancer images, 379 esophagitis images, and 193 normal images. The validation set was an independent test set used to evaluate the diagnostic performance of the MTCS model, prepared from 64 patients with 57 cancer images, 94 esophagitis images, and 47 normal images. It is worth mentioning that the training set and validation set were selected from two independent groups of patients aged 23 years or older, and the ratio of males to females was approximately 1:1. The workflow diagram of dataset collection is shown in Figure 1.

All images in our dataset were captured using conventional endoscopes with standard WLI and NBI. Standard single-accessory channel endoscopes (GIF-Q240Z, GIF-RQ260Z, GIF-FQ260Z, GIF-H260Z, GIF-Q260J GIF-H290Z, GIF-HQ290, and GIF-XP290N, Olympus, Tokyo, Japan) were used in this study. To ensure the correctness of each image in the datasets, the criteria for identifying either abnormal or normal esophageal images were confirmed by both the preliminary endoscopy report and pathological results and reviewed by at least two experienced physicians. All esophageal lesions in the images were then marked manually by experienced endoscopists who had on average over 10 years of experience in endoscopy operations.

### 2.2. Development of the MTCS Model

**Figure 2:**
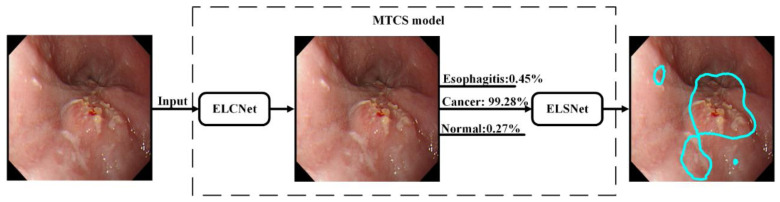
The diagnostic procedure of the MTCS model.

We proposed a novel MTCS model consisting of ELCNet and ELSNet for the classification and segmentation of esophageal lesions using esophageal endoscopic images. Difficulty in distinguishing the representative features among multiple esophageal lesions may prevent deep learning models from accurately segmenting the lesions, thus it is of great significance to design a lesion-specific segmentation task after the model has classified the type of lesions automatically in order so that the first task of the MTCS model was the multiple classifications of esophagus lesions that separate the images into three categories, including “cancer”, “esophagitis”, and “normal” using ELCNet, and then the second task was to perform the segmentation through learning the shared features for a specific lesion such as cancer using ELSNet. Among them, the dilated convolution was used in the ELSNet to improve its performance. The diagnostic procedure of the MTCS model is shown in Figure 2. Finally, we measured the performance of MTCS, and the endoscopists tested it.

#### 2.2.1. Design of ELCNet and ELSNet

**Figure 3:**
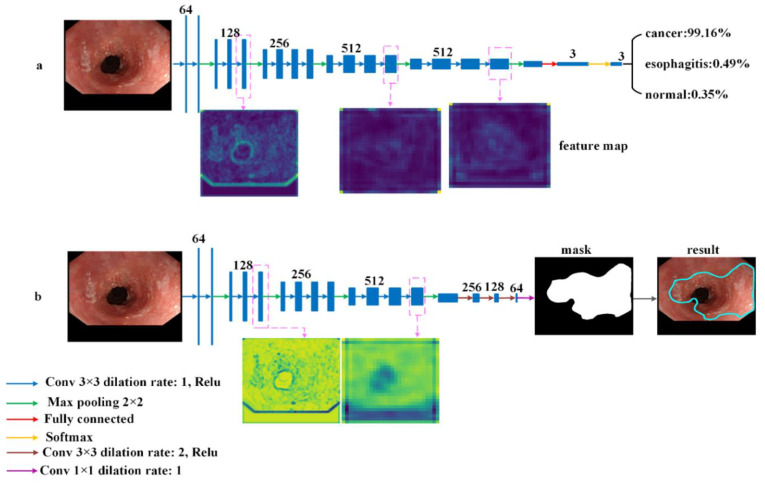
(**a**) Architecture of ELCNet; (**b**) architecture of ELSNet.

For classification, we proposed the neural network, ELCNet, based on a typical deep learning VGG-16 model [28]. To improve the training efficiency to be better adapted to our dataset, we compressed the fully connected layer of the original model to reflect the scale of our datasets. Therefore, the ELCNet was composed of 13 convolutional layers, five max-pooling layers, and one fully connected layer. The architecture of the ELCNet is shown in Figure 3a.

For the segmentation task, we proposed an ELSNet for esophageal lesion segmentation. The ELSNet was modified with an end-to-end asymmetric structure, in which the downsampling of the ELSNet was composed of ten convolutional layers and three dilated convolutional layers, while the upsampling of the ELSNet was implemented by bilinear interpolation to ensure that the output resolution was the same as that of the input image and retained better features in the output image. More importantly, we designed a dilated convolution method [29] in ELSNet. It has an advantage in extracting more useful features and increasing the resolution of the image, thereby it can further improve the performance of segmentation. The dilated convolution was defined as follows:

(1) $$y(m,n)=\sum_{i=1}^{M}\sum_{j=1}^{N}x(m+r\times i,\;n+r\times j)w\left(i,j\right)$$

where *y*(*m*, *n*) is the output, *x*(*m*, *n*) is the input, *w*(*i*, *j*) is the filter with the *M* length and *N* width, and *r* is the dilation rate. It should be noted when *r* is equal to 1, the dilated convolution is the same as normal convolution. In our work, considering the trade-off between model segmentation performance and computational complexity, we set the r rate size to 2. The architecture of the ELSNet is shown in Figure 3b.

No matter what ELCNet or ELSNet, the cross-entropy loss function is used as the loss function. It is defined by:

(2) $$L_{loss}=-\frac{1}{K}\sum_{k=1}^{K}\left(g_{k}\log(p_{k})+(1-g_{k})\log(1-p_{k})\right)$$

where *K* is the number of datasets, *g* is the truth label, and *p* is the output of the ELCNet or ELSNet.

#### 2.2.2. Evaluation Metric of ELCNet and ELSNet

To quantitatively analyze the performance of the proposed models, we employed the following four different metrics.

First, the main outcome measures were diagnostic accuracy, sensitivity, specificity, positive predictive value (PPV), negative predictive value (NPV), and computational complexity. We used these measures to estimate the diagnostic performance of the MTCS model with the validation set and to evaluate how endoscopists can improve their performance when using the MTCS model. They are defined as:

(3) $$\mathrm{Accuracy}=\frac{\sum_{c=1}^{C}\left({\mathrm{TP}}_{c}+{\mathrm{TN}}_{c}\right)}{\sum_{c=1}^{C}\left({\mathrm{TP}}_{c}+{\mathrm{TN}}_{c}+{\mathrm{FP}}_{c}+{\mathrm{FN}}_{c}\right)}\times 100\%$$

(4) $$\mathrm{Sensitivity}=\frac{1}{C}\sum_{c=1}^{C}\frac{{\mathrm{TP}}_{c}}{{\mathrm{TP}}_{c}+{\mathrm{FN}}_{c}}\times 100\%$$

(5) $$\mathrm{Specificity}=\frac{1}{C}\sum_{c=1}^{C}\frac{{\mathrm{TN}}_{c}}{{\mathrm{TN}}_{c}+{\mathrm{FP}}_{c}}\times 100\%$$

(6) $$\mathrm{PPV}=\frac{1}{C}\sum_{c=1}^{C}\frac{{\mathrm{TP}}_{c}}{{\mathrm{TP}}_{c}+{\mathrm{FP}}_{c}}\times 100\%$$

(7) $$\mathrm{NPV}=\frac{1}{C}\sum_{c=1}^{C}\frac{{\mathrm{TN}}_{c}}{{\mathrm{TN}}_{c}+{\mathrm{FN}}_{c}}\times 100\%$$

where *C* is the number of types of esophageal lesions. True positives (TP) means the number of positive samples is correctly classified. True negatives (TN) means the number of negative samples is correctly classified. False positives (FP) means the number of negative samples is wrongly classified as positive. False negatives (FN) means the number of positive samples is wrongly classified as negative.

Second, we used the receiver operating characteristic (ROC) curve to show the diagnostic performance of classification. ROC curves are created by plotting the proportion of the true-positive rate against the proportion of the false-positive rate by varying the predictive probability threshold. The true-positive rate is equal to sensitivity, and the false-negative rate can be obtained by 1-specificity. A larger area under the curve (AUC) indicates better diagnostic performance.

Third, we used the confusion matrix to analyze the classification performance on each category of esophageal lesion on ELCNet. Each column of the confusion matrix indicates the predicted categories, and the total number of each column represents the number of images predicted to be that type; each row indicates the true categories of the images, and the total number of images in each row represents the number of images of that type. The value in each column indicates the number of real images predicted to be of that type.

Finally, a dice similarity coefficient (DSC) and an intersection over union (IoU) were used to evaluate the image segmentation performance. The DSC represents the degree of overlap between the ground truth region and segmented region, and the IoU represents the ratio of the intersection and union of the ground truth region and segmented region. The larger they are, the better the segmentation performance. They are defined as:

(8) $$\mathrm{DSC}=\frac{2|X\cap Y|}{\left|X|+|Y\right|}\times 100\%$$

(9) $$\mathrm{IoU}=\frac{\left|X\cap Y\right|}{\left|X\cup Y\right|}\times 100\%$$

where *X* represents the ground truth, which is masked by endoscopists, and *Y* is the segmentation region of the proposed model.

## 3. Experiments and Results

In order to improve the performance of the MTCS model using a smaller dataset, we proposed a pre-trained VGG-16 model on ImageNet for our networks, which adopted an SGD optimizer with a batch size of 8, a learning rate of 1×10^4^, and the largest epoch was 100. Moreover, we also used data augmentation to reduce the risk of overfitting, including crop, flip, rotation, and color jitter so that the number of training images was expanded to five times the original training dataset. Other compared methods set the same parameters as the proposed model, and their pre-trained model is also based on ImageNet if using the pre-trained model. All methods used the same set of images we collected to get the experimental results.

All methods were implemented using the PyTorch platform (1.6.0) on the environment: Ubuntu 16.04.5, Python 3.7, and GTX1080TI.

### 3.1. Comparison of the MTCS Model and Other Methods

When using the validation set to evaluate the classification performance of the MTCS model, we compared the proposed model with other methods, including VGG-16 used by Liu et al. [19], AlexNet used by Igarashi et al. [20], GoogLeNet used by Kumagsi et al. [21], ResNet-50 used by Zhu et al. [22], and ECA-Net proposed by Wang et al. [30]. We firstly calculated accuracy, sensitivity, specificity, PPV, NPV, and computational complexity for all methods. As can be observed from Table 2 the classification performance of the ELCNet outperformed other methods on accuracy (93.43%), sensitivity (92.82%), specificity (96.20%), PPV (94.25%), and NPV (96.62%). These values were higher than the lowest indices (Zhu et al. [22]) by 3.87%, 8.81%, 4.72%, 9.57%, and 4.87% and more than the suboptimal indices (Liu et al. [19]) by 1.51%, 4.34%, 2.74%, 6.06%, and 3.18%. The parameter amount of the ELCNet was only 14.79 M, which was slightly more than that of Kumagsi et al. [21], and less than that of other methods.

**Figure 4:**
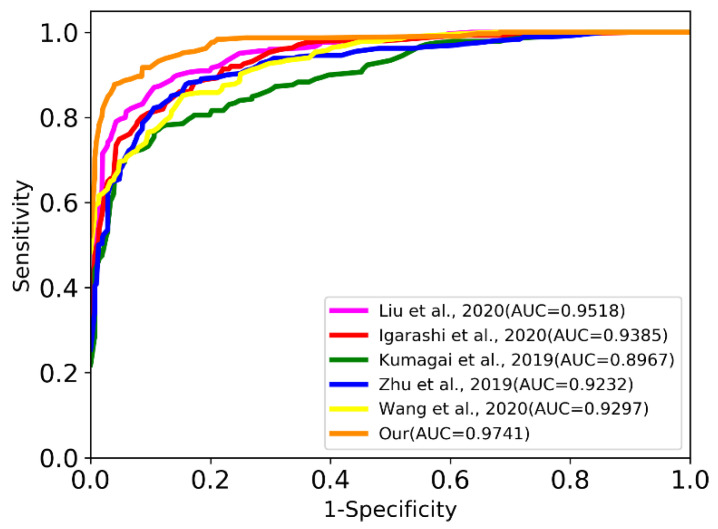
Receiver operating characteristic of ELCNet and other methods.

As can be seen from Figure 4 the ROC curve of the ELCNet was a better performance than that of other methods. Specifically, the AUC of the ELCNet was 0.0774 higher than the lowest value (Kumagai et al. [21]) and more than the suboptimal value (Liu et al. [19]) by 0.0223.

**Figure 5:**
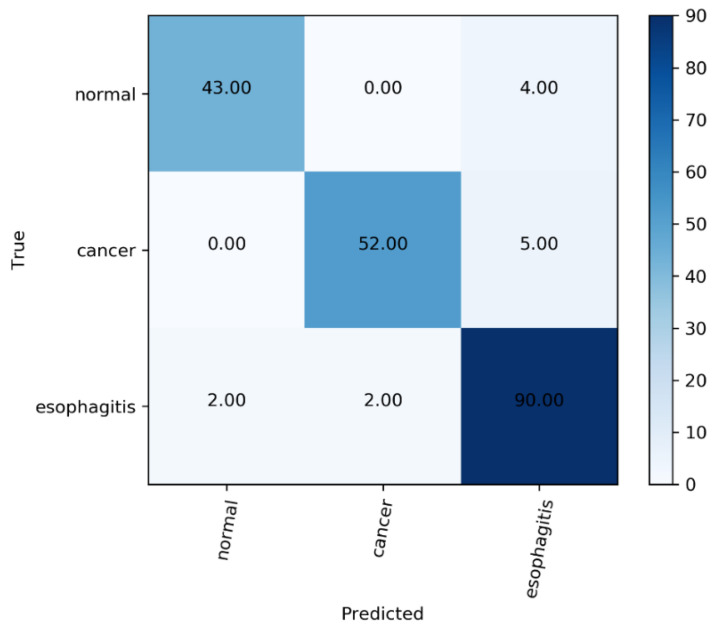
The confusion matrix of ELCNet.

In addition, the confusion matrix of ELCNet, as shown in Figure 5, intuitively indicated that most esophageal lesions (cancer: 52/57, esophagitis: 90/94, and normal: 43/47) could be classified into correct categories of lesions by ELCNet.

**Figure 6:**
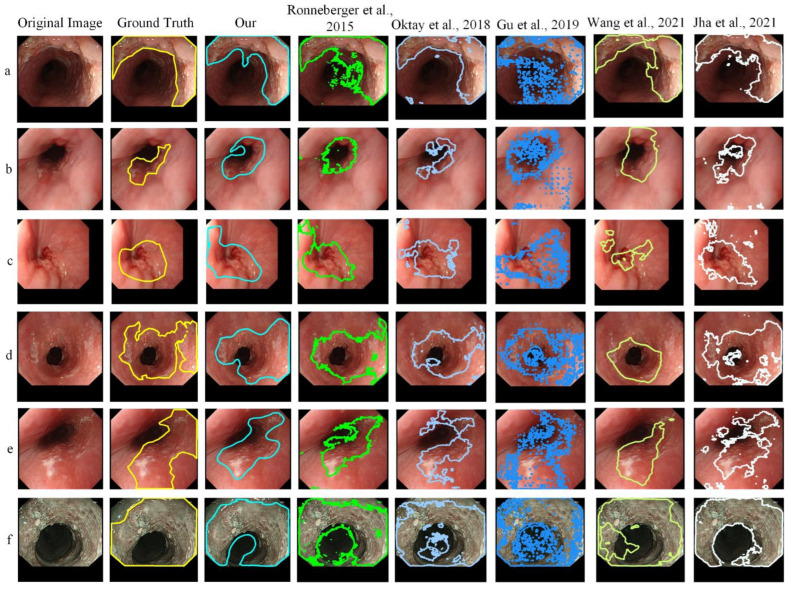
(**a**–**f**) Comparison of cancer segmentation between ELSNet and other methods.

When comparing the segmentation performance of the MTCS model with other conventional symmetric networks, including U-Net proposed by Ronneberger et al. [31], Attention U-Net proposed by Oktay et al. [32], CE-Net proposed by Gu et al. [33], HRNet proposed by Wang et al. [34], and ColonSegNet proposed by Jha et al. [35] on the validation set. Figure 6 shows that the marked area of different cancer types can achieve satisfactory results, and the ELSNet achieved better results than other methods in cancer segmentation. In our study, the cancer type mainly includes esophageal squamous cell carcinoma and esophageal adenocarcinoma.

We can see from Table 3 that the ELSNet achieved the highest values on DSC (77.84%) and IoU (65.63%) and surpassed that of Gu et al. [33] by 2.02% and 3.50%, respectively. Moreover, the parameter amount of the proposed method was only 9.18 M, which was 4.17 M more than that of Jha et al. [35], and less than half of that of other methods.

Finally, we calculated the training time of the proposed MTCS. The average training time of each epoch of ELCNet was 10.06 s, the average training time of each epoch of ELSNet was 13.25 s, and each network performed 100 epochs, respectively. The training time of ELCNet and ELSNet was 16.77 min and 22.08 min, respectively. Therefore, the training time of the MTCS was 38.85 min by adding the two subnetwork training times.

### 3.2. Comparison between the MTCS Model and Endoscopists

The endoscopists who participated in the testing included senior (endoscopy experience > 10 years) and junior (endoscopy experience < 10 years) endoscopists and the ratio was approximately 1:1. Before participating in the test, these endoscopists were not involved in the labeling of the lesions and had no access to the validation set. Accurate classification of the categories of esophageal diseases is essential for performing a lesion-specific segmentation. The results of the classification performance of the MTCS model compared with the endoscopists are shown in Table 4. The accuracy, sensitivity, specificity, PPV, and NPV of the MTCS model were higher than those of the endoscopists. Compared with the performance of the endoscopists, the accuracy, sensitivity, specificity, PPV, and NPV increased by 9.59%, 13.92%, 8.30%, 17.84%, and 9.17%, respectively.

Based on these favorable results, the MTCS model not only had a good diagnostic performance for diagnosing esophageal lesions but also was relatively lightweight and consumed fewer computing resources. Hence, the proposed model could help endoscopists minimize errors without questioning their diagnostic abilities. Additionally, the final diagnosis always has to be confirmed by them.

### 3.3. Ablation Studies

In this subsection, we analyzed the contribution of using a pre-trained model and dilated convolution on the performance of the proposed model. Table 5 shows the performance of the ELCNet whether to use a pre-trained model. Compared with the model without the pre-trained model, the accuracy, sensitivity, specificity, PPV, and NPV of the ELCNet are improved 4.03%, 3.99%, 2.28%, 3.71%, and 2.16%, respectively.

Furthermore, as can be observed from Table 6 the performance of the ELSNet is improved by using a pre-trained model and dilated convolution, boosting 3.28% DSC and 4.89% IoU.

## 4. Discussion

Several methods for screening esophageal lesions have been developed in the past few decades and are mainly based on endoscopic technology [36,37,38]. Although advanced endoscopic equipment has improved the diagnosis of esophageal lesions, only experienced endoscopists can recognize the subtle differences between different lesions. Therefore, the overall shortage of well-trained endoscopists is a major problem worldwide [39], causing them to be busy reading a large number of images every day to filter out the images with lesions.

To solve this problem, it is necessary to develop a new method by extracting the features of the images for automatically judging and annotating different esophageal lesions. Traditional feature extraction methods based on machine learning [40,41] are usually employed for medical images. However, these methods have the disadvantages of feature extraction and selection being time-consuming and vary according to different types [42].

Moreover, earlier deep learning methods for medical image segmentation were mostly based on image patches. For example, Ciresan et al. [43] presented segment neuronal membranes in microscopy images based on patches and a sliding window strategy. However, these methods have two main disadvantages: redundant calculations caused by sliding windows and the inability to learn global features. In recent years, the success of the fully convolutional network [44] was witnessed, which is an end-to-end network in image processing, and it was proved that the end-to-end network was a popular neural network architecture for biomedical image segmentation tasks [23,45,46].

Based on these findings, we proposed a novel MTCS model, which was composed of two relatively independent subnetworks (ELCNet and ELSNet), to improve the diagnostic accuracy of endoscopists with the support of the predicted classification on multiple types of lesions and the suggested segmentation on each specific type of lesion. Specifically, we reduced the fully connected layers in the ELCNet to improve classification performance and used the dilated convolution in the ELSNet to increase segmentation performance. Based on the dataset (cancer, esophagitis, and normal images) we collected, the proposed model can achieve higher performance in esophageal lesion classification and can completely annotate the cancer region with a higher DSC and IoU compared with other methods in esophageal lesion analysis. Since there is currently no publicly available data set on the esophagus, we cannot compare the MTCS with other methods on other esophagus datasets, but we believe it can achieve satisfactory performance if other similar esophagus datasets are composed of cancer, esophagitis, and normal images are available. Therefore, we succeeded in developing the MTCS model that can classify multiple esophageal lesions and segment-specific lesions based on standard WLI and NBI. In practical terms, when endoscopists use the MTCS model to perform assist examinations on the input esophageal endoscopic images, the MTCS model will display predicted results such as the type and lesion region of the input image to the endoscopists and assist them in making the final diagnosis decision.

According to the needs of tasks in practical applications, we need to combine two relatively independent subnetworks (ELCNet and ELSNet) in serial order as an MTCS model. Therefore, for the input esophageal endoscopic image, the proposed MTCS first used ELCNet to classify it into one of three categories (cancer, esophagitis, and normal) and used ELSNet to segment lesion regions when the lesion type is predicted to be cancer. Therefore, the main advantage of the proposed MTCS is that it can achieve better performance in identifying esophageal lesions from endoscopic images since their subnetworks are trained separately to reduce feature interference between subnetworks. On the contrary, the disadvantage is that when the ELC classification is wrong, such as other lesions are predicted to be cancer or cancer is predicted to be other lesions, the ELS segmentation will also mis-segment the lesions or miss the diagnosis.

Additionally, there are already existing commercial systems (such as GI Genius, Medtronic) that are mainly used for the detection of only one lesion (colorectal polyps). Compared with these commercial systems, the proposed model focuses on classifying and segmenting esophageal lesions. Furthermore, the segmentation task of the proposed model can locate the cancer lesion area. It is better than the detection-based methods since it avoids the problem of inaccurate positioning but high confidence.

Our study has several limitations. First, the sources of our datasets were only from Macau Kiang Wu Hospital, although it is the most representative hospital in Macau, the sample size was small. Therefore, we plan to collect esophageal images from more hospitals and centers in future research and include more endoscopists from more hospitals and centers to participate in our research. Second, our work only focused on cancer and esophagitis and did not include other esophageal lesions such as esophageal polyps, esophageal leiomyoma, and esophageal hernia. These esophageal lesions will be considered in the future. Third, since the labeling of endoscopic images takes time, we will consider using limited labeled datasets and a large number of unlabeled datasets to develop a semi-supervised or self-supervised network.

## 5. Conclusions

In this paper, we proposed the MTCS model, including ELCNet and ELSNet, to realize the classification and segmentation for esophageal lesions. To improve our model for classifying and segmenting multiple lesions, the ELCNet compressed the fully connected layer to increase the training efficiency, and the dilated convolution was designed to extract more useful features and increase the resolution of the image in ELSNet. Compared with other related methods, the proposed model not only distinguished multiple esophageal lesions (cancer, esophagitis, and normal) with higher accuracy, sensitivity, specificity, PPV, and NPV on image-level classification but also outputted the shape, size, and texture of esophageal lesions (cancer) on pixel-level segmentation with higher DSC and IoU. Additionally, when compared with the endoscopist, these values increased by 9.59%, 13.92%, 8.30%, 17.84%, and 9.17%, respectively. Based on these favorable results, the MTCS model is an effective and efficient computer-aided diagnostic tool for analyzing multiple esophageal lesions.

## Figures and Tables

**Table 1:** Comparison of the methods for esophageal lesion analysis.

Methods	Tasks	Dataset	Image Type	Performance
Liu et al. [16]	Classification	Private	Endoscopic image	85.83% accuracy
Du et al. [17]	Classification	Private	Endoscopic image	90.63% accuracy
Liu et al. [19]	Classification	Private	Endoscopic image	89.00% accuracy
Igarashi et al. [20]	Classification	Private	Endoscopic image	96.5% accuracy
Kumagsi et al. [21]	Classification	Private	Endoscopic image	90.90% accuracy
Zhu et al. [22]	Classification	Private	Endoscopic image	89.16% accuracy
Wang et al. [18]	Segmentation	Public	Endoscopic image	74.00% intersection over union
Huang et al. [23]	Segmentation	Private	Computed tomography	72.55% dice similarity coefficient
Chen et al. [24]	Segmentation	Private	Computed tomography	79.00% dice similarity coefficient
Zhou et al. [25]	Segmentation	Private	Computed tomography	84.839% dice similarity coefficient
Yousefi et al. [26]	Segmentation	Private	Computed tomography	79.00% dice similarity coefficient

**Table 2:** Comparison of the classification performance of our model and other methods.

Methods	Pre-Trained	Accuracy	Sensitivity	Specificity	PPV	NPV	Parameters	FLOPs
Liu et al. [19]	yes	91.92%	88.48%	93.46%	88.19%	93.44%	134.27 M	123.84 G
Igarashi et al. [20]	yes	91.59%	87.06%	92.92%	88.02%	93.26%	57.02 M	5.69 G
Kumagai et al. [21]	no	91.92%	88.71%	93.54%	87.89%	93.39%	6.30 M	209.45 G
Zhu et al. [22]	yes	89.56%	84.01%	91.48%	84.68%	91.75%	23.51 M	32.87 G
Wang et al. [30]	no	90.91%	86.00%	92.45%	86.87%	92.71%	21.29 M	29.38 G
Our	yes	93.43%	92.82%	96.20%	94.25%	96.62%	14.79 M	122.88 G

**Table 3:** Comparison of the segmentation performance of our model and other methods.

Methods	Pre-Trained	DSC	IoU	Parameters	FLOPs
Ronneberger et al. [31]	No	75.11%	61.84%	31.04 M	875.49 G
Oktay et al. [32]	No	75.78%	62.34%	34.88 M	1.065 T
Gu et al. [33]	Yes	75.82%	62.13%	29.00 M	142.60 G
Wang et al. [34]	No	74.31%	60.96%	29.53 M	362.60 G
Jha et al. [35]	No	75.31%	61.71%	5.01 M	993.96 G
Our	Yes	77.84%	65.63%	9.18 M	317.38 G

**Table 4:** Diagnostic performance of the MTCS model and the endoscopists.

Performance	Accuracy	Sensitivity	Specificity	PPV	NPV
Our	93.43%	92.82%	96.20%	94.25%	96.62%
Endoscopists	83.84%	78.90%	87.90%	76.41%	87.45%

**Table 5:** The performance of the ELCNet with and without a pre-trained model.

Pre-Trained	Accuracy	Sensitivity	Specificity	PPV	NPV	Parameters	FLOPs
Yes	93.43%	92.82%	96.20%	94.25%	96.62%	14.79 M	122.88 G
No	89.40%	88.83%	93.92%	90.54%	94.46%	14.79 M	122.88 G

**Table 6:** The performance of the ELSNet with and without a pre-trained model and dilated convolution.

Pre-Trained	Dilated Convolution	DSC	IoU	Parameters	FLOPs
Yes	Yes	77.84%	65.63%	9.18 M	317.38 G
Yes	No	76.14%	62.48%	9.18 M	298.34 G
No	No	74.56%	60.74%	9.18 M	298.34 G

## Data Availability

The data supporting reported results are available on request from the corresponding author. The data are not publicly available due to the Macau law for the privacy of patients.
